# Effects of therapeutic goal management (TGM) on treatment attendance and drug abstinence among men with co-occurring substance use and axis I mental disorders who are homeless: results of the Birmingham EARTH program

**DOI:** 10.1186/1940-0640-8-17

**Published:** 2013-10-27

**Authors:** Anna Davidson, Michael Jensen, Emilee Burgess, Angee Stevens, Lauren Hayes, Susan Sieweke, Karen Stough, Anne Wright, Robin McCarty, Lillian Eddleman, Young-il Kim, Jesse B Milby, Joseph E Schumacher

**Affiliations:** 1 Department of Psychology, University of Alabama at Birmingham, Birmingham, AL, USA; 2 Division of Preventive Medicine, University of Alabama at Birmingham, 1717 11th Avenue South, MT 616, Birmingham, AL 35205, USA; 3 Fire House Shelter, Birmingham, AL, USA; 4 Jefferson, Blount, St. Clair Mental Health Authority, Birmingham, AL, USA; 5 Church of the Reconciler, Birmingham, AL, USA

**Keywords:** Substance abuse treatment, Severe mental illness, Homelessness, Therapeutic goal management

## Abstract

**Purpose:**

This study describes the implementation and impact of Therapeutic Goal Management (TGM) in a Substance Abuse and Mental Health Services Administration (SAMHSA)-sponsored demonstration project entitled Enhanced Addiction Recovery through Housing (EARTH).

**Participants:**

The sample included 28 male participants followed at six months who completed some treatment. Forty-three percent were Caucasian, and 57% were African American. The average age of participants was 42 years.

**Design:**

The relationships between TGM goal achievement, treatment attendance, and drug abstinence outcomes were studied among EARTH program participants who were homeless and met criteria for co-occurring substance use and severe DSM-IV Axis I mental disorders.

**Results:**

The results revealed an overall drug abstinence rate of 72.4% over six months and significant positive relationships between TGM goal achievement and drug abstinence (r = 0.693) and TGM goal achievement and treatment attendance (r = 0.843).

**Conclusions:**

This research demonstrated the relationship and potential positive impact of systematically setting, monitoring, and reinforcing personalized goals in multiple life areas on drug abstinence and treatment attendance outcomes among persons who are homeless with co-occurring substance use and other Axis I disorders in a integrated community service delivery program.

## Background

Use of goal setting and task motivation has been studied for over 35 years. Locke and Latham [1] reviewed an extensive literature on goal theory, mechanisms through which goals have their effects, moderators of goal effects, goals as mediators for incentives, and the relationship of goals to satisfaction. A current application of goal setting in the field of behavioral treatment of drug addiction is Therapeutic Goals Management (TGM).

The TGM goal-setting intervention is driven by behavioral principles and person-centered, recovery-oriented treatment philosophy. The theoretical foundation for this reinforcement approach to drug addiction is based on a Skinnerian psychological model of behavior change, generally referred to as applied behavior analysis. Contemporary behavioral theories of reinforcement are also based on models of choice [2-4]. The person-centered approach to recovery [5] consists of reorientation from patient to personhood, reorientation of what is considered valued knowledge and expertise, and partnership and negotiation in decision-making. The premise of TGM is that preference for any particular reinforcer (e.g., drug use) will depend on the more general context of access to other reinforcers.

Human studies [6,7] suggest that a carefully structured treatment program, which systematically exposes people who abuse drugs to new sources of nondrug-related reinforcers in a nondrug using social context, may be a viable treatment approach to cocaine dependence. Successful models of applied behavior analysis for effectively treating drug and alcohol dependence have been demonstrated. They include contingency management [8,9], contingency-managed housing and work therapy [10-14], voucher-based reinforcement [15-17], behavioral activation [18], and other behavioral therapies [19].

The evidence documenting TGM as an effective behavioral treatment for cocaine dependence and homelessness is based on four randomized controlled trials [10-14]. This research demonstrated that persons with primary crack cocaine addiction who are homeless can be effectively retained and treated and achieve positive drug abstinence and housing outcomes using innovative contingency managed housing, work therapy (in some studies), and behavioral day treatment with TGM goal-setting.

We describe the implementation and impact of TGM in a US Department of Health and Human Services (DHHS) Substance Abuse and Mental Health Services Administration (SAMHSA)-sponsored translational research project entitled Enhanced Addiction Recovery through Housing (EARTH). The EARTH program was a coordinated effort combining three community agencies to provide treatment for homelessness, drug addiction, and severe mental illness through integrated contingency managed housing, behavioral day treatment, and psychiatric case management driven by TGM. The relationship between TGM, treatment attendance, and drug abstinence outcomes was studied among persons who were homeless and met criteria for co-occurring substance use and severe DSM-IV Axis I mental disorders.

## Methodology

### Description of the research context

The Birmingham metropolitan area is a city of over one million citizens, among whom approximately 2929 persons are homeless on any given day. Eighty-two percent of people in this group have been homeless less than two years. It was estimated that 35% live in shelters or on the street, 29% are chronically homeless, 30% have drug and/or alcohol use disorders, and 18% have serious mental disorders [20]. The EARTH program was designed to improve personal outcomes related to drug addiction, homelessness, and mental illness among dually diagnosed persons in the Birmingham area.

The EARTH program is a coordinated effort between three community agencies under the direction of investigators from the University of Alabama at Birmingham (UAB) Division of Preventive Medicine (DOPM) and Department of Psychology. Birmingham’s Firehouse Shelter provides safe and accessible shelter and contingency managed (transitional) housing, case management, and 12-Step support groups. The Jefferson, Blount, and St. Clair Mental Health Authority (JBS) provides psychiatric outreach, case management, and pharmacotherapy for people who are homeless and serves as the recruitment site for EARTH at the downtown Birmingham First Light Shelter. Finally, the Church of the Reconciler (COR) is the site for the behavioral day treatment program and provides transportation, meals, and application support for disability insurance and Housing and Urban Development (HUD) shelter-plus-care permanent housing.

EARTH is the result of two decades of research on effective treatments for persons who are homeless conducted by Milby and Schumacher [10-14]. This research was the first to use contingency management to treat cocaine addiction and homelessness by providing access to programs that provided housing (and work therapy in some studies) contingent on drug abstinence. In EARTH, drug-abstinence-contingent housing is coupled with a behavioral day treatment program that uses TGM to set, monitor, and reinforce personal goal achievement in multiple life areas. Life areas are usually defined as satisfaction with housing status, addiction, employment/income and constructive use of free time, nondrug-related social and recreational activities, and health/mental health. The EARTH program offers comprehensive evidence-based interventions for drug and alcohol addiction and severe mental illness to persons who are homeless through an integrated community partnership.

### Population and recruitment

Participants in the EARTH program were recruited from the Birmingham metropolitan area through the JBS psychiatric and homelessness outreach program. Male adults who met criteria for (1) McKinney Act [21] definition of homelessness, (2) drug and/or alcohol dependency (DSM-IV substance use disorders criteria), and (3) severe mental illness [22] were eligible for enrollment. The McKinney Act defines the term “homeless”, “homeless individual”, and “homeless person” to mean (1) an individual or family who lacks a fixed, regular, and adequate nighttime residence; (2) an individual or family with a primary nighttime residence that is a public or private place not designed for or ordinarily used as a regular sleeping accommodation for human beings, including a car, park, abandoned building, bus or train station, airport, or camping ground; (3) an individual or family living in a supervised publicly or privately operated shelter designated to provide temporary living arrangements (including hotels and motels paid for by federal, state, or local government programs for low-income individuals or by charitable organizations, congregate shelters, and transitional housing); (4) an individual who resided in a shelter or place not meant for human habitation and who is exiting an institution where he or she temporarily resided; (5) an individual or family who will imminently lose their housing, including housing they own, rent, or live in without paying rent, are sharing with others, and rooms in hotels or motels not paid for by federal, state, or local government programs for low-income individuals or by charitable organizations [21].

Severe mental illness diagnoses include major depression, schizophrenia, bipolar disorder, obsessive compulsive disorder (OCD), panic disorder, post-traumatic stress disorder (PTSD), and borderline personality disorder [22]. Persons who met eligibility criteria and who were interested in participating were scheduled for an orientation and informed-consent interview with the EARTH research assistant. Research and program evaluation procedures were approved for human subject’s protection by The University of Alabama at Birmingham Institutional Review Board.

### Design and hypotheses

A prospective correlational design was used to assess the relationships between TGM goal achievement, treatment attendance, and drug abstinence outcomes over the six-month EARTH treatment period. It was hypothesized that TGM goal achievement would be positively associated with treatment attendance and drug abstinence rates for participants over the course of treatment.

### EARTH procedure and intervention

Interested persons were oriented to the EARTH project, provided with informed consent, and enrolled. Enrolled participants were administered a battery of psychological assessments at treatment entry for purposes of developing the initial TGM Plan, program evaluation, and pre-post treatment outcome comparisons. For purposes of this paper, the Mini-International Neuropsychiatric Interview (MINI), version 5.0 for DSM-IV, was used to assess Axis I substance use disorders and other mental disorders [23].

Enrolled participants were rapidly provided with contingency-managed housing through Firehouse, which included transportation to their own furnished apartment shared with one to two roommates. Participants had access to program-provided housing during the six-month treatment period and beyond if interested. Participants engaged in behavioral day treatment consisting of TGM, breakfast and lunch, transportation, and other psychosocial interventions at COR. There were two phases: the maximum number of days in Phase I was 60, and in Phase II was 24, for a total of 84 days. Psychiatric case management and pharmacotherapy was provided through regular appointments with a psychiatrist located at the downtown JBS site. Achievement of TGM goals, treatment attendance, and drug abstinence (measured by twice weekly urine drug toxicology tests) were monitored during treatment. Finally, the baseline assessment battery was repeated after completion of six months of treatment. Refer to SAMHSA’s National Registry of Evidence-based Programs and Practices (NREPP) for more details about Behavioral Day Treatment (including TGM) and Contingency Managed Housing and Work Therapy [24].

### Therapeutic goal management (TGM) procedure

Therapeutic Goals Management (TGM) is a person-centered behavior therapy for treating drug and alcohol addiction and co-occurring mental disorders and enhancing functioning in multiple areas of life. It is a goal-setting strategy based on a Skinnerian psychological model of behavior change and an extensive literature on goal theory. The goal-setting theory strategy includes mechanisms through which goals have their effects, moderators of goal effects, goals as mediators for incentives, and the relationship of goals to satisfaction. The development, implementation, and evaluation of TGM by Drs. Jesse B. Milby and Joseph E. Schumacher over the past two decades have been sponsored by the National Institute on Drug Abuse (NIDA), the National Institute on Alcohol Abuse and Alcoholism (NIAAA), and the Substance Abuse and Mental Health Services Administration (SAMHSA). Online training for TGM was developed through SAMHSA (TGM eLearning Course) and online implementation of TGM is available through *ChipRewards, Inc*. (Meta CM—TGM).

The purpose of TGM is to improve functioning in multiple life areas, reduce hazardous drug use and negative consequences associated with drug use, and improve problems in life functioning caused by drug use. It elicits and negotiates the development of person-centered goals through a facilitation process between the therapist and participant rather than simply providing expert advice or a standardized treatment plan. As opposed to focusing entirely on problems or weaknesses, TGM focuses on strengths. Perceived ideals (long-term goals) are defined, and personal strengths and resources are identified to empower the participant to take specific, realistic, and measurable steps (short-term goals) to accomplish long-term goals.

Therapeutic Goal Management is a behavioral intervention. Goals are developed using applied behavioral analysis, and progress toward and accomplishment of goals is monitored weekly through specific, measurable, and objective criteria. Goal achievement is reinforced through positive peer recognition and modest monetary reinforcement. Plans for TGM are strategically developed and updated at multiple treatment transition time points or phases. The Phase I (treatment entry) TGM plan is for three months; the Phase II or aftercare TGM plan covers the next three months (for a total length of six months); and Phase III or the final TGM plan is designed for an indefinite period of time to be used after the completion of EARTH.

There are four structured, integrated, procedural components of TGM: Initial Goal Development, Goal Review, Goal Development, and Goal Reward. Initial Goal Development is a one-on-one negotiation process between a trained TGM therapist and the participant. It is designed to jumpstart the TGM process and finalize an initial TGM plan.

The process of Initial Goal Development utilizes personalized feedback from baseline assessment information and a structured quality-of-life interview to assist the participant in defining long-term and short-term goals in five life areas in the EARTH program (housing, addiction, employment, recreation, and health). The TGM therapist elicits and facilitates, rather than directs or advises, the participant to define the nature of dissatisfaction (rather than problems) with addiction, housing, work, recreation, and health areas of his or her life and identify specific, realistic, and measurable goals to achieve an ideal state of satisfaction in each life area. An example of a person’s long-term goal for addiction is, “Define what my personal drug-free lifestyle consists of by the end of Phase I”. For each long-term goal, the therapist and participant then collaborate on defining two to three short-term verifiable goals that can be accomplished during the coming weeks. An example of one short-term goal would be, “I will write a two-page self-disclosure statement of my drug-use history in my journal by next Tuesday (with date) as evidenced by reading it to the group in Community Morning Meeting”. Short-term goals usually address who, what, how, when, and documentation questions. Most participants will have at least 15 short-term goals as a result of the Initial Goal Development process.

Goal Review (and Reward) and Goal Development components are conducted weekly, either one-on-one or in a group setting facilitated by a TGM therapist. Goal Review begins with each participant reading their TGM Plan goals to the group, providing objective evidence (“as evidenced by”) for goal achievement, rating their own goal accomplishment (e.g., met and on time, partially met, not met, no opportunity, etc.), confirming goal accomplishment rating through peer agreement (TGM therapist resolves disputes), and receiving social reinforcement (e.g., clapping, shout-outs, “Boom!”, pats on the back, raising the roof gestures) and tangible rewards (cash incentives) for goal achievement. In EARTH, rewards of $5 cash for 80 to 99% goal achievement and $10 cash for 100% goal achievement are provided weekly. In Goal Development, participants update their TGM Plan by taking turns reviewing them and adding, modifying, or deleting goals that are achieved, unrealistic, or not under the control of the participant. This process is designed to maintain a proper challenge in number and rigor of goals for each individual participant. A complete TGM administration manual is available from the authors and through NREPP.

### Case example

The following is an excerpt from a hypothetical but typical EARTH participant, fictitiously named “Willie Nedson”. Provided here are sample long- and short-term goals for Willie in Addiction and Recreation life areas (italics indicate the participant’s own goal narrative).

Willie is a 35-year-old African-American man who was referred to the EARTH program by the program’s outreach component. Willie is unemployed and living in a community homeless shelter for people. He has been using alcohol and crack cocaine on a daily basis and has co-occurring mental disorders. He is a skilled welder, has military experience, and has family support from his parents living in the area. He was recently discharged from the service because of psychological problems. Willie expressed interest in learning more about the EARTH program and was scheduled for an orientation, assessment, and an Initial TGM Plan (Phase I) interview for a two- to three-month long-term goal completion endpoint.

Upon completion of Willie’s Initial TGM Plan, his goals for the Addiction life area were as follows: First, his long-term goal for addiction was *Learn strategies to stop using crack cocaine by the end of Phase I treatment*. His short-term goals for this area were *1) Learn and practice one relapse-prevention skill each week for a total of eight skills (as evidenced by) passing a role-play test of one relapse prevention skill each Friday agreed by my therapist and peers; 2) Make a list of pros and cons of using crack cocaine (as evidenced by) sharing pros and cons of using crack cocaine by reading one pro and one con from my journal each Monday during Community Morning Meeting; 3) Submit a urine sample for drug testing two times each week (as evidenced by) the drug-testing technologist who will record my test results and chart on a graph.*

Willie’s long-term goal for the Recreation life area was *Run a six-minute mile by the end of three months*. His short-term goals were *1) Eat five servings of fruits and vegetables a day by making a daily menu and shopping for enough fresh fruits and vegetables for a week at a time (as evidenced by) initialing each consumed fruit or vegetable daily from my menu; 2) Buy a watch with a timer by using my first $10 incentive during Wednesday shopping trip (as evidenced by) wearing my new watch and demonstrating timer in Goal Review Group next Friday; 3) Find an exercise partner who lives in my apartment complex by announcing at a house meeting that I am looking for an exercise partner and putting up a sign on laundry room board (as evidenced by) partner will sign my journal stating he/she is joining me or coaching my daily exercising within one week from today; 4) Increase my distance and improve my time running by running every morning, recording the distance and timing myself (as evidenced by) recording my runs in my journal with initials by my partner or coach.*

### Measurement and variables

Goal achievement in TGM was measured during weekly goal review groups. Each week during the six month behavioral day treatment period, long- and short-term goals were reviewed for progress by peers and a TGM therapist for each participant. Each short-term goal was read aloud to the group with a report of documented objective evidence of progress called “as evidenced by”. The participant then rated his progress on a scale of 1 (not at all completed) to 4 (100% completed on time) with “no opportunity” or “goal not rated this day” options not counted against him. After agreement by peers or resolution by the TGM therapist, the percentage of goals fully achieved and on time (rating of 4) out of the total number of short-term goals for that participant (typically around 15 short-term goals) was calculated and ranged from 0% to 100% for each of the 24 weeks of EARTH. This weekly calculation was logged, charted on the participant’s TGM Plan, and recorded on a data collection form. The TGM goal-achievement variable was the average of the weekly percentage of goals achieved across 24 weeks.

Treatment attendance was documented as present or absent (unexcused) for a full day (10 am to 2 pm) of behavioral day treatment Monday through Friday for Phase I for three months or 12 weeks (total possible attendance = 60 days) and Tuesday and Wednesday for Phase II for three months or 12 weeks (total possible attendance = 24 days) (range, 0 to 84 possible days of attendance). Excused absences, including doctor visits, food-stamp office appointments, and case manager appointments, were considered therapeutic and counted as present.

Drug abstinence was measured using the on site drug ProScreen Drugs of Abuse screening test from US Diagnostics. Participant urine samples were screened for benzodiazepines, cocaine, methamphetamines, opiates, and THC or their metabolites. Urine samples were obtained under observation from a same sex research assistant for testing on Monday and Thursday of each week for Phase I (12 weeks for a total possible of 24 tests) and Tuesday or Wednesday for Phase II (12 weeks for a total possible of 12 tests). Positive or negative screening test results for each substance were recorded. A negative result was assigned on each test day during the six-month treatment period if all substances screened negative (range, of 0 to 36), and a positive result was assigned if any substance was positive. The percentage of negative tests (drug abstinence) was calculated by dividing the number of negative tests out of the total number of tests administered for a range of 0 to 100% negative. An unexcused missing test was scored the same value as a positive.

### Analyses

Frequencies, averages, and distributions of TGM goals and goal achievement, treatment attendance, and drug abstinence variables were calculated. The relationships between TGM goal achievement, treatment attendance, and drug abstinence were analyzed by calculating Pearson r correlations.

## Results

### Participant characteristics and follow-up and rate

For this study, 28 participants had the opportunity to complete the six months of EARTH treatment. All 28 were male (100%), 16 were African American (57%), and 12 were Caucasian (43%). They ranged in age from 18 to 58 years with an average age of 42 years. Length of homelessness among participants ranged from six months to more than three years. Employment history ranged from no formal employment to positions requiring doctoral degrees, and substance use in the past 30 days ranged from some to daily. All participants completed a follow-up assessment at six-months post-treatment entry for a follow-up rate of 100%.

### Substance use and other mental disorders

Tables 1 and 2 present the types and prevalence of DSM-IV Axis I substance use disorders and other mental disorders, respectively. While all participants admitted to recent substance abuse, 82% of the participants met criteria for at least one substance use disorder at baseline. Cocaine dependence and alcohol dependence were the most common diagnoses. For other mental disorders, 93% met criteria for at least one other mental disorder, of which bipolar I disorder and post-traumatic stress disorder were the most common. All participants had a history of at least two psychiatric hospitalizations in their past.

**Table 1:** Prevalence of substance use disorder diagnoses

**Substance use disorder**	n (%)*
Cocaine dependence	14 (43%)
Alcohol dependence	10 (30%)
Alcohol abuse	4 (12%)
Opioid dependence	2 (6%)
Cannabis abuse	1 (3%)
Inhalant dependence	1 (3%)
Cannabis dependence	1 (3%)

*82% of participants had at least one SUD.

**Table 2:** Prevalence of other mental disorders

**Other mental disorders**	n (%)*
Bipolar I disorder	17 (28%)
Post-traumatic stress disorder	14 (23%)
Panic disorder (any)	15 (9%)
Major depression (any)	8 (13%)
Psychotic disorder (not otherwise specified)	5 (8%)
Generalized anxiety disorder	3 (5%)
Social phobia	1 (2%)
Agoraphobia	1 (2%)
Obsessive compulsive disorder	1 (2%)
Bipolar II disorder	1 (2%)

*93% of participants had at least one mental disorder.

### Drug testing, treatment attendance, and goal achievement

The total number of drug tests administered over six-month treatment period was 894, with a range of six to 54 tests per participant and an average of 30.6 tests per participant (which represented 85% of the maximum number of drug tests available per participant). A few participants were tested more than the scheduled allotment at their request or for other administrative reasons. The mean proportion of negative (drug-abstinent) test results for the overall sample was 72.4% (SD = 34.7). All participants completed 65.7% of possible days of treatment during the six-month treatment period. Twenty-three participants (79%) completed at least 30% and 21% completed less than 30% of the treatment days available. Participants who completed more treatment, as defined above, were significantly more abstinent (mean = 85.9%, SD = 24.6) than their counterparts (mean = 23.0%, SD = 15.0) (t-test value = 5.91, p < 0.0001). They did not differ from those who completed less than 30% of treatment days with regard to homelessness, drug addiction, or severity of mental illness at treatment entry. There was an average of 15 goals reviewed per participant with 92% rated goal met and on time for the total sample.

### Relationship between TGM goal achievement and drug abstinence and treatment attendance variables

**Figure 1:**
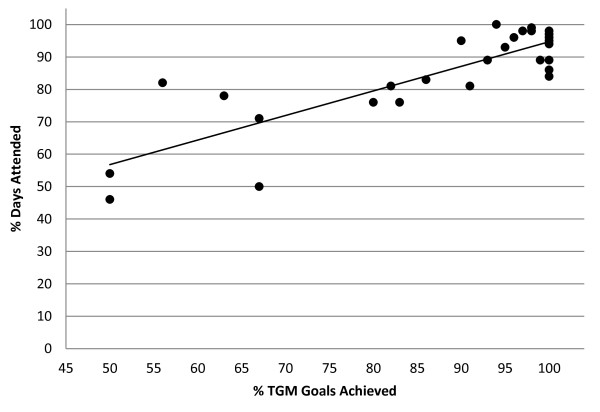
Scatter plot of mean percentage of days attended by percentage of TGM goals achieved.

**Figure 2:**
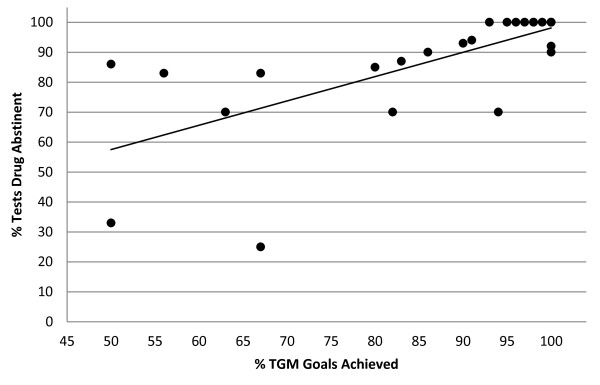
Scatter plot of mean percentage of tests that were abstinent for drugs by percentage of TGM goals achieved*.

The relationship between TGM goal achievement and drug abstinence and TGM achievement and treatment attendance are presented in Figures 1 and 2, respectively.

Figure 1 is a scatter plot of mean percentage of days attended by percentage of TGM goals achieved. The Pearson r correlation between these two variables was r = 0.843 (p < 0.0001). Figure 2 is a scatter plot of mean percentage of tests drug abstinent by percentage of TGM goals achieved. The Pearson r correlation between these two variables was r = 0.693 (p < 0.001).

## Discussion

This study described and tested the impact of TGM in a community-based collaborative homelessness, addiction, and severe mental illness support program. The EARTH program offered comprehensive evidence-based interventions for drug and alcohol addiction and severe mental illness to persons without homes through an integrated community partnership. Contingency managed housing was coupled with a behavioral day treatment program that used TGM to set, monitor, and reinforce personal goal achievement in multiple life areas. Results revealed strong positive relationships between TGM goal achievement and drug abstinence and TGM goal achievement and treatment attendance. These findings suggest that TGM has a positive impact on treatment exposure and drug abstinence outcomes in this population. This study demonstrated high overall compliance rates of goal setting, goal achievement, attendance, and drug abstinence outcomes.

This study represents a continuation of research by the authors using behavioral day treatment (including TGM) and contingency-managed housing interventions to treat cocaine addiction among person without homes. While most of the studies included persons with non-psychotic mental disorders, this study was the first to include integrated community services and treatment for persons with serious mental illnesses. The drug abstinence rates found at six months in this homeless population with serious mental illnesses (72.4%) were higher than, but close to, rates found using the same intervention among persons with nonpsychotic mental disorders in a meta-analysis of four controlled trials (57%) [13]. Similar findings of greater improvements in five of the seven life areas on the Addiction Severity Index among participants with dual mood or anxiety disorders were found in an earlier comparison of behavioral day treatment and contingency management interventions [25]. This study also shows greater rates of abstinence among participants who complete more treatment and is consistent with our previous research that more intensive contact early in treatment results in better long-term outcomes amone people with cocaine abuse [26]. Finally, several review studies support the generalizability and efficacy of coordinated treatment programs for homeless adults [27], incentive-based treatments [28], psychosocial interventions including contingency management [29], and assertive community treatment [30] for reducing drug use among people with co-occurring substance use disorders and severe mental illness.

This study describes the procedure for generating rich, person-centered goals in five major life areas in a day addiction-treatment context. Participants in EARTH regularly reported that drug toxicology testing and TGM were the two most important components of the behavioral day treatment experience. The personalized goal-setting process was intensive, and most participants were surprised that they could set anywhere from 15 to 20 realistic short-term goals within a few days of admission. Weekly social and monetary reward for goal achievement was highly valued by participants and likely responsible for attendance on Goal Review Group days. Attendance on other days was reported to be motivated by not wanting to miss the opportunity to accomplish goals and report goal progress that influences their reward potential. Goal achievement was also designed to impact drug abstinence in two ways. First, goals were set specifically for drug use behavior and relapse prevention in the Addiction life area. Second, several more goals were set in other life areas that are theorized to influence drug use, like recreation. It appears that the TGM intervention in this behavioral day treatment context is attractive and motivating to participants with co-occurring substance use and other mental disorders who are homeless, and is associated with positive treatment attendance and drug abstinence outcomes.

Limitations of this study include small sample size and nonrandomized TGM treatment comparison research design. Goal achievement, treatment attendance, and drug abstinence variables tended to be skewed toward the high end of the range and lacked variability. A predictable limitation was the potential confounding or tautological effect on the attendance and drug abstinence outcomes of some participants actually having short-term goals identical to the outcome. For example, some participants set goals to have negative drug toxicology tests or attend day treatment groups. To control for this, we eliminated any such goals from these analyses. After removal of such goals, only six participant’s overall goal achievement outcome percentage variable changed. Finally, given that the goal setting process is individualized and the TGM Plans are unique to each participant, it is impossible to tell which goals or sets of goals were responsible for the relationship with attendance and drug abstinence outcomes. Along with this personalization, the process of goal setting, goal review, and goal reward itself appeared to be the combined driving force behind the impact. Future research should focus on using a more controlled experimental design to determine the effects of TGM on important personal outcomes and the differential effects of life areas.

The strengths of this study include assessment of highly structured goal setting, goal monitoring, and goal reward procedures. The TGM intervention is delivered by therapists using manual-driven scripts and worksheets. Therapists undergo training by Drs. Milby and Schumacher using didactic, role-playing, and observation techniques. Trained TGM therapists are observed and monitored periodically for fidelity and adherence to the manual.

Another strength of this study is the rigor and objectivity of outcome measurement. Both attendance and drug abstinence were objectively measured and represent common and important outcomes in the drug addiction treatment literature. Measurement of TGM goal achievement also shares qualities of objectivity and consistency in rating goal achievement. The detailed process of rating goals is an additional strength. It included documenting hard evidence of goal-meeting outcomes for each short-term goal in the participant’s TGM plan; providing evidence to peers and the TGM therapist in the weekly Goal Review Group (for example, a movie ticket stub as opposed to self-report); self-rating of the goal achievement levels (eg, fully met and on time, fully met but not on time, partially met, or not met) with peer group and TGM therapist agreement; and resolution of disputes by the TGM therapist. Finally, the successful demonstration of the TGM process and its relationship to attendance and abstinence outcomes was among a complex, dually diagnosed, and basic-need-compromised population. The multidimensionality (for example, setting goals in multiple life areas) and personalization aspects of TGM fit well with the characteristics of this population and likely contributed to attractiveness, motivation, and impact of this intervention.

## Competing interests

The authors declare that they have no competing interests.

## Authors’ contribution

AD, MJ, EB, AS, LH, JM, and JS contributed conceptually, analytically, and writing of the manuscript and implementation of the intervention tested; SS, KS, AW, RM, and LE contributed to the implementation of the intervention tested; YK contributed to the statistical analysis and preparation of tables and figures; and all authors read and approved the final manuscript.
